# Deep neural network analysis models for complex random telegraph signals

**DOI:** 10.1038/s41598-023-37142-9

**Published:** 2023-06-27

**Authors:** Marcel Robitaille, HeeBong Yang, Lu Wang, Bowen Deng, Na Young Kim

**Affiliations:** 1 Institute for Quantum Computing, University of Waterloo, 200 University Ave W, Waterloo, ON N2L 3G1 Canada; 2 Department of Electrical and Computer Engineering, University of Waterloo, 200 University Ave W, Waterloo, ON N2L 3G1 Canada; 3 Waterloo Institute for Nanotechnology, University of Waterloo, 200 University Ave W, Waterloo, ON N2L 3G1 Canada; 4 Department of Chemistry, University of Waterloo, 200 University Ave W, Waterloo, ON N2L 3G1 Canada; 5 Perimeter Institute, University of Waterloo, 31 Caroline St N, Waterloo, ON N2L 2Y5 Canada

**Keywords:** Electrical and electronic engineering, Computational science

## Abstract

Time-fluctuating signals are ubiquitous and diverse in many physical, chemical, and biological systems, among which random telegraph signals (RTSs) refer to a series of instantaneous switching events between two discrete levels from single-particle movements. A reliable RTS analysis is a crucial prerequisite to identify underlying mechanisms related to device performance and sensitivity. When numerous levels are involved, complex patterns of multilevel RTSs occur and make their quantitative analysis exponentially difficult, hereby systematic approaches are often elusive. In this work, we present a three-step analysis protocol via progressive knowledge-transfer, where the outputs of the early step are passed onto a subsequent step. Especially, to quantify complex RTSs, we resort to three deep neural network architectures whose trained models can process raw temporal data directly. We furthermore demonstrate the model accuracy extensively with a large dataset of different RTS types in terms of additional background noise types and amplitude size. Our protocol offers structured schemes to extract the parameter values of complex RTSs as imperative information with which researchers can draw meaningful and relevant interpretations and inferences of given devices and systems.

## Introduction

**Figure 1:**
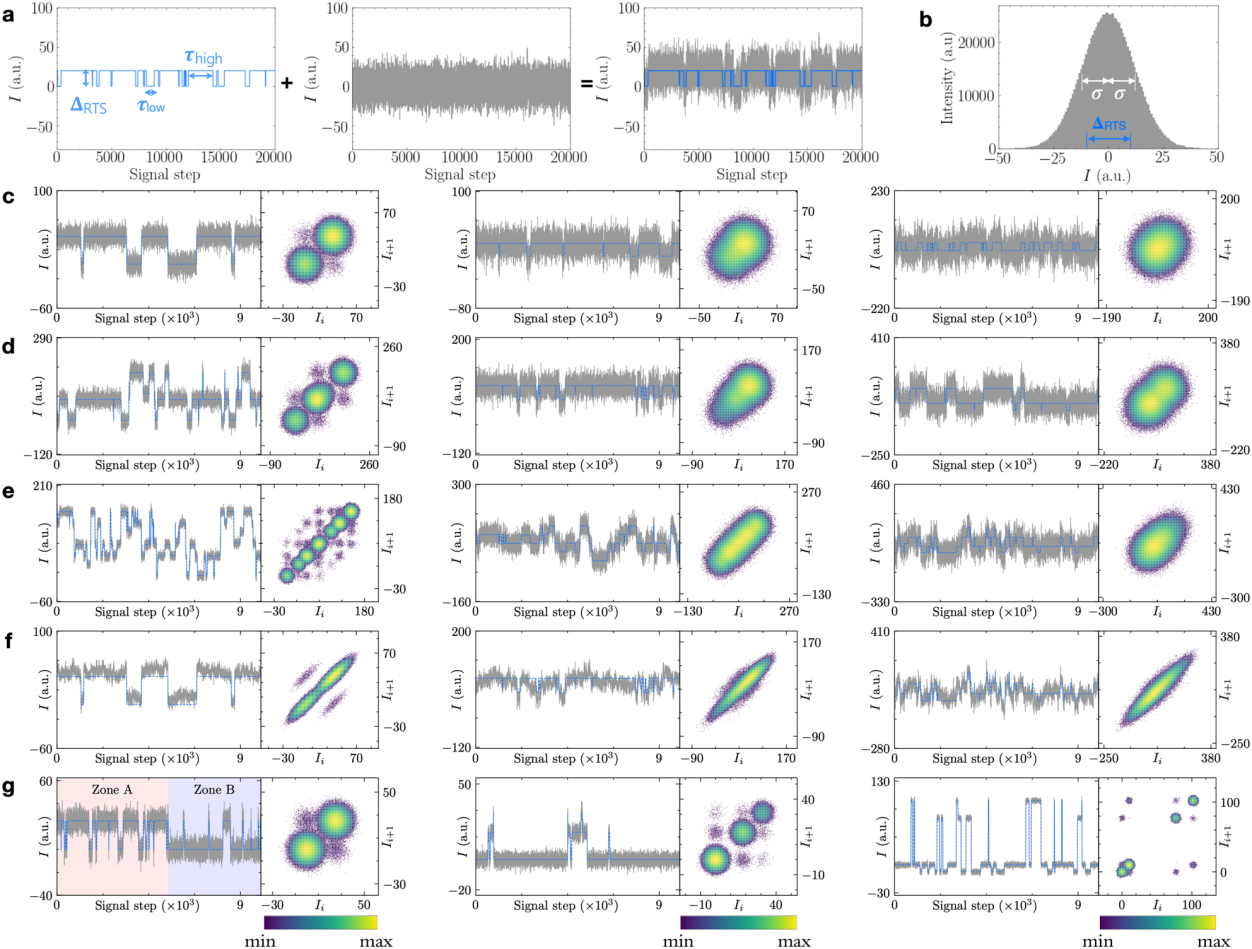
Types of synthetic random telegraph signals. (**a**) A representative 1-trap noisy signal (right) is synthesized by summing random telegraph signals (left) and background white noise with $\sigma$ = 20% (middle). (**b**) The white noise amplitudes in the example of (**a**) obey the Gaussian distribution with standard deviation $\sigma$ = 20% of the $\Delta _\text {RTS}$. (**c**–**f**) The time-series and time-lag plots for exemplary signals from normal random telegraph signals (nRTSs) of (**c**) 1-trap, (**d**) 2-trap, and (**e**) 3-trap with Gaussian white noise levels $Q_\text {wn}={20}{\%}$ (left), $Q_\text {wn}={60}{\%}$ (middle), and $Q_\text {wn}={100}{\%}$ (right). The definition of $Q_\text {wn}$ is given in “Methods” Section. (**f**) Representative nRTSs with 1/*f* (pink) noise in place of white noise for 1-trap with $Q_\text {wn}=$20% pink noise (left), 2-trap and 60% pink noise (middle), 3-trap and 100% pink noise (right). (**g**) The time-series and time-lag plots for exemplary signals with three anomalous RTS types: a metastable signal with the two distinctive zones (A and B) annotated and color-coded (left), a missing-level signal where it is possible to see only 3 clusters on the diagonal of the time-lag plot (middle), and a coupled signal where it is possible to see that the amplitude of the fast trap changes with the state of the slow trap (right). The blue digitized signals are obtained from the Bi-GRU model in all time-domain signals.

Random telegraph signals (RTSs) are sequential data that comprise a time series of burst signals between two well-defined states. As a fundamental type of low-frequency stochastic fluctuations^1–4^, RTSs appear in a wide variety of disciplines: physics, chemistry, biology, and engineering. Two-level RTSs are manifestations of a single-carrier stochastic movement that can switch abruptly between high and low levels separated by $\Delta _\text {RTS}$ with individual characteristic dwell times $\tau _{\text {high}}$ and $\tau _{\text {low}}$ defined in a representative signal (Fig. 1a), whose mean values of $\bar{\tau }_\text {high}$ and $\bar{\tau }_\text {low}$ obey Poisson statistics^5^. These distinctive RTSs are frequently seen in small-sized electronic devices such as metal-oxide-semiconductor field-effect transistors^6–9^, resistive random access memory devices^10^, memristors^11,12^, and complementary metal-oxide semiconductor (CMOS) image sensors^13^. For example, in a submicron-sized metal-oxide-semiconductor field-effect transistor (MOSFET), if there is a trap to capture or emit a single charge carrier, device currents in the transport channel exhibit arbitrary patterns of two-level RTSs over time. Similar RTS phenomena are discerned in superconducting qubits^14,15^, single photon avalanche diodes^16^, ultrasensitive biosensors^17^, and single-cell activities in ion channels^18^. Real-time dynamics in quantum electrical devices also reveal rapid jumping sequences^19,20^. Furthermore, these bursting events can represent biological and biochemical processes like spontaneous transitions in gene-regulation networks^21,22^, where RTS models are adopted to explain stochastic individual gene activation and deactivation processes well. Therefore, a thorough analysis of RTSs is essential to quantify the performance sensitivity of devices and to deepen the fundamental understanding of systems and processes in diverse areas.

Tremendous efforts have been put into establishing RTS analysis methods that obtain quantitative values of three parameters $\Delta _\text {RTS}$, $\bar{\tau }_\text {high}$, and $\bar{\tau }_\text {low}$ for each RTS. A basic way to determine $\Delta _\text {RTS}$ assesses a histogram of all data points; a clear bimodal distribution in the histogram confirms the two discrete levels of RTS, consequently giving its $\Delta _\text {RTS}$ value. $\bar{\tau }_\text {high}$ and $\bar{\tau }_\text {low}$ are computed as the statistical mean of each time duration from digitized signals to represent RTSs. A common barrier to estimate RTS parameters accurately is another unwanted noise such as white Gaussian noise or pink noise, which masks the RTSs (Fig. 1a, b). In order to overcome this barrier, a time-lag plot (TLP) has been exploited to depict not only level-corresponding peaks (diagonal) but also between-level transitions (off-diagonal)^23^, and a weighted TLP further improves peak-detection efficiency by minimizing unwanted broad noise^24^. A wavelet denoising method from conventional signal processing techniques was attempted to analyze RTS noise in CMOS image sensors^25^. The discrete wavelet transform (DWT) with the Haar wavelet allows the suppression of background white noise. The reconstruction process of a digitized RTS consists of seven-stage denoising routines after trial and error to clean the background noise. In order to remove incorrect transitions, additional steps of the temporal screen and second thresholding in the DWT were adopted. Unfortunately, this protocol works for a two-level RTS and it suffers from hit-and-miss errors^25^. Another common denoising technique in image and signal processing analysis, partial differential equation (PDE), examines stochastic processes and their behavior in both spatial and time domains^26–30^. Recently, there is an attempt to utilize a PDE to *n*-independent RTS noises along a real space where an indiscernible origin of the RTS process^31^. On the other hand, our analysis focuses on a single RTS sequence and establishes a robust and systematic way to reconstruct a digitized trace only by recognizing random and instantaneous signal-switching events in the time domain, which cannot directly be studied with the PDE method. Notwithstanding the mixture of RTS and other background noise, a simple hidden Markov model (HMM) has analyzed two-level RTSs by assigning a pair of two-by-two matrices to represent hidden layers associated with transition probabilities between the states. HMM performs remarkably well in obtaining $\Delta _\text {RTS}$ and digitizing the signals into the binary levels despite flip-errors, which can be readily corrected^32–34^.

As the physical size of devices further shrinks down to nanoscales or movements of a single particle play important roles, complex RTS patterns among multiple discrete levels of several traps have often been captured^35,36^. Figure 1c–g showcase representative noisy signals with two-level and multi-level RTSs. We include the TLP plots of each example, which visualize the autocorrelation maps of the one-dimensional time traces to identify RTS states from diagonal clusters and the transitions between the states from off-diagonal data points^23^. As the number of RTS-levels increases, the total number of parameters to determine each RTS surges exponentially, where the simple HMM fails to estimate all parameters successfully because digitizing noisy signals into multiple discrete levels is extremely challenging. Hence, more advanced factorial HMMs (fHMMs) with many hidden layers were proposed and attempted to evaluate multi-level RTSs, maximizing its capacity to handle complex sequential data^37–39^.

**Figure 2:**
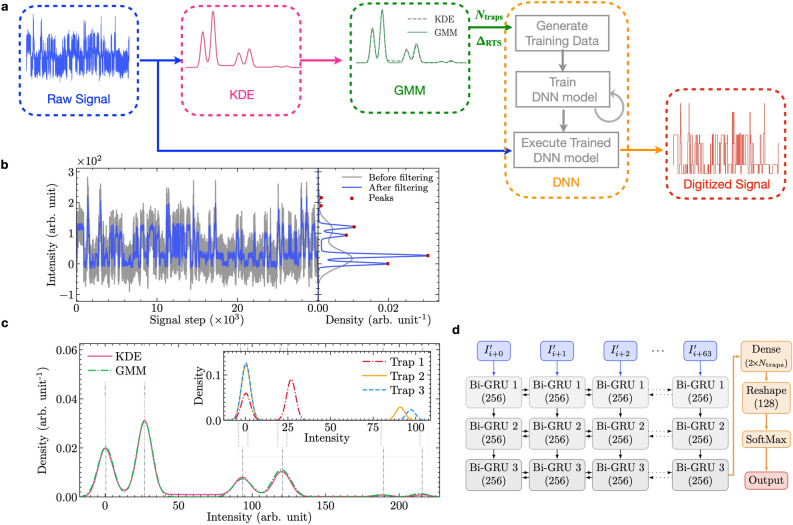
A flow chart of a three-step RTS analysis protocol and exemplary results of Step 1 and Step 2. (**a**) A flowchart representing the flow of data through our algorithm with visualizations for all the three steps. (**b**) Raw (gray) and filtered (﻿﻿﻿blu﻿e) time-series of 3-trap RTSs with $Q_\text {wn}={20}{\%}$ (left). The distributions of RTSs before (gray) and after (blue) filtering by kernel density estimate (KDE) method in Step 1. (**c**) A Gaussian mixture model (GMM) in green is fitted to the KDE to extract individual trap distributions and amplitudes, where ground truth center values are shown with dotted gray vertical lines. The inset shows the decomposed density of three individual traps. (**d**) The structure of our recurrent neural network, where Bi-GRU is the standard bidirectional gated recurrent unit from Keras.

Although fHMMs assess complex RTSs well, to set up a fHMM is not straightforward but daunting due to the lack of knowledge to initialize all values of many matrix elements that define the number of hidden layers to construct the fHMM. Worst, the number of all matrix elements rises steeply as more discrete levels are involved. Furthermore, few attempts have been made to perform model validation through extensive statistical analysis of RTS parameters with a large data set. There is one work^35^ that evaluated full parameters using Bayesian inference only for eight multi-trap RTS examples; however, it has limited statistical analysis of accuracy and a lack of structured study regarding additional background noise^35^. Recently, two interesting studies attempted to build neural network models for examining RTSs. One study applied a clustering neural network to assess a long-time series of currents in a resistive random access memory device with improved weighted TLP^40^, and the other work constructed a long short-term memory (LSTM) recurrent neural network (RNN) to evaluate phase RTSs in superconducting double dots^15^. Hence, systematic analysis models of complex RTSs are sought-after, and to the best of our knowledge, a step-by-step protocol for the quantitative RTS analysis and its explicit descriptions are still needed. Here we demonstrate a three-step RTS analysis protocol (Fig. 2a) based on several machine learning (ML) algorithms and deep learning architectures (See “Methods”). As far as we know, we validate the RTS analysis protocol thoroughly for the first time with a large synthetic dataset (720 examples whose individual time trace is 1,000,000 long, Fig. 1c–g) that include various types of RTS signals deteriorated by two different background noise classes (white and pink noise) whose degradation impact is widely varied in 11 different levels. Real current signals from carbon nanotube (CNT) devices and *n*-channel metal-oxide semiconductor (NMOS) devices are further analyzed by our protocol.

## Results

The first two steps determine two quantities of the trap number ($N_\text {traps}$) and $\Delta _\text {RTS}$ that become initial values to generate large training data together with featureless background noise for mimicking raw signals in practice. Figure 2b and c display representative results of Step 1 kernel density estimate (KDE) and Step 2 Gaussian mixture model (GMM). These generated data are utilized to train deep neural network (DNN) models built upon three advanced architectures that are specifically designed to process sequential data: (1) Bi-directional gated recurrent unit (Bi-GRU)-based recurrent neural networks (RNNs), (2) Bi-directional long short-term memory (Bi-LSTM)-based RNNs, and (3) WaveNet convolutional neural networks (CNNs)^41^. Figure 2d illustrates a representative RNN architecture with Bi-GRUs. As the flowchart indicates in Fig. 2a, we emphasize that the trained DNN models execute directly the original raw signals (blue in Fig. 2a) to produce digitized signals (red in Fig. 2a), wherein our novelty lies.

In order to acquire the statistical validation of our RTS analysis protocol and to compare the execution performance among three different DNN architectures, we synthesize a multitude of simulated signals, each having 1,000,000 time steps in total. $N_\text {traps}$ in the simulated signals selects 1, 2, and 3. Consequently, the maximum associated number of discrete RTS levels are 2, 4, and 8, respectively. Moreover, we consider normal and anomalous RTSs. The former results from the superposition of multiple ($N_\text {traps}$) independent two-level RTSs (Fig. 1c–f), whereas the latter exhibits complex interaction schemes between traps, namely, the traps are not independent in the anomalous case (Fig. 1g). In addition to variations on simulated the RTS itself, we add two types of background noise: Gaussian white noise and pink noise, that deteriorate the underlying RTS and impede the analysis. In the following, we present the results from the algorithm performance validation study on synthetic normal RTS (nRTS) and anomalous RTS (aRTS) with multiple background noise sizes. Moreover, we examine real RTSs from short-channel CNT film devices and NMOS devices.

The performance results are summarized quantitatively in terms of the RTS amplitude ($\Delta _\text {RTS}$) and two averaged time constants ($\bar{\tau }_\text {high}$ and $\bar{\tau }_\text {low}$) whose error is calculated by,

1 $$\begin{aligned} \epsilon (X)&\equiv \frac{|{X}_\text {theory} - {X}_\text {estimate}|}{{X}_\text {theory}} \times {100}{\%}, \end{aligned}$$

where *X* represents each of three RTS parameters ($\Delta_\text {RTS}$, $\bar{\tau }_\text {high}$, $\bar{\tau }_\text {low}$), ${X}_\text {theory}$ is the design value for parameters of the simulated RTSs, and ${X}_\text {estimate}$ is the output from our algorithm analysis. Note that the theoretical values of $\bar{\tau }_\text {high}$ and $\bar{\tau }_\text {low}$ are evaluated from the statistical analysis of the actual synthetic RTSs. The digitization ($\eta$) error is obtained from the direct comparison of the time traces, the background-noise-free RTSs and the digitized RTSs at each time. Any mismatch at a given time is counted as the digitization error.

### Validation on synthetic normal random telegraph signals with white noise

**Figure 3:**
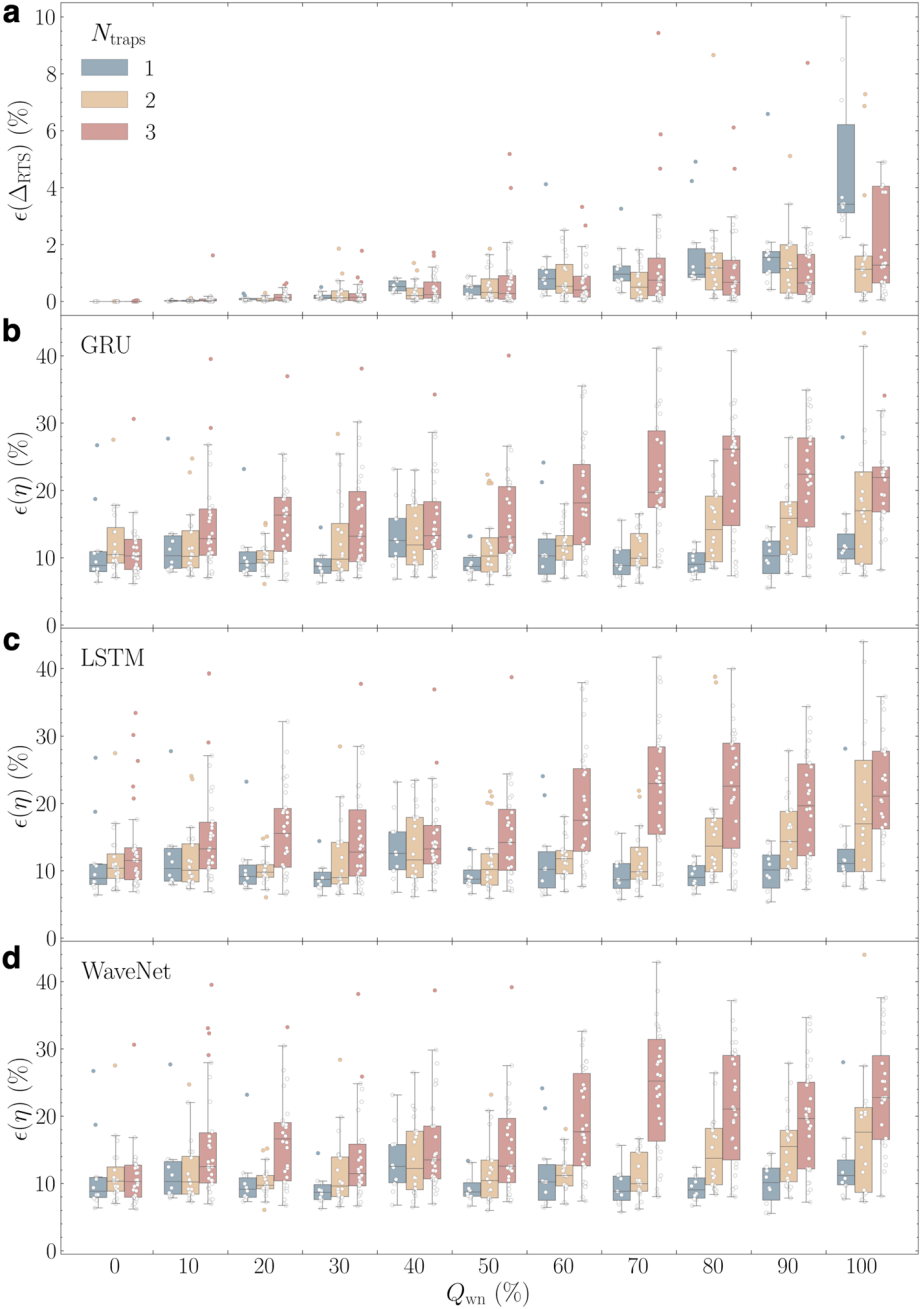
Validation results of nRTSs with white noise background. (**a**) $\epsilon (\Delta _\text {RTS})$, the error in predicting $\Delta _\text {RTS}$ from the KDE and GMM steps are plotted on a linear scale. The digitization error $\epsilon (\eta )$ is computed by three trained deep neural network models. (**b**) Bi-directional gate recurrent units (Bi-GRU). (**c**) Bi-directional long short-term model (Bi-LSTM); (**d**) WaveNet for $N_\text {traps}=$ 1 (blue), 2 (yellow), and 3 (red) examples. Note that each point corresponds to individual RTS, so in each $Q_\text {wn}$ bin, the boxplot of $N_\text {traps}=$ 1 contains 10 RTS pattern examples, but those of $N_\text {traps}=$ 2 and 3 are from 20 and 30 RTS patterns. We also added individual data points as circles on top of boxplots that allow us to present outliers explicitly.

First, we apply our three-step analysis protocol to all 330 nRTSs synthesized for $N_\text {traps}\in \{1,2,3\}$ that are veiled by 11 sizes of the background white noise amplitudes, $Q_\text {wn}\in \{0\,\%,10\,\%,20\,\%,\dots , 100\,\%\}$ (See “Methods”). Figure 3 collects all error analysis results over the entire dataset. The KDE step followed by the second GMM step estimates $N_\text {traps}$ and $\Delta _\text {RTS}$, with which the amplitude error $\epsilon (\Delta _\text {RTS})$ is calculated. Our algorithm detects $N_\text {traps}$ correctly for all 110 examples (100 % success) where $N_\text {traps}\in \{1,2\}$, but only for 107 of the 110 (97.3 % success) 3-trap RTS examples. The three failed examples of 3-trap RTS occur when the amplitudes of two traps are very close and the difference of these amplitudes is easily obscured by background noise, which can hamper the recognition of individual peaks.

We choose boxplot graphs on a linear scale to present explicitly the distribution of $\epsilon (\Delta _\text {RTS})$ along $Q_\text {wn}$, giving median and interquartile range for $N_\text {traps}$—1 (blue), 2 (yellow), and 3 (red)—side by side in Fig. 3a. As $N_\text {traps}$ becomes higher and $Q_\text {wn}$ escalates, it is increasingly difficult to estimate accurate $\Delta _\text {RTS}$ values, resulting in higher $\epsilon (\Delta _\text {RTS})$. Indeed, the difficulty of this task grows exponentially with $N_\text {traps}$ due to the increased number of KDE peaks (RTS levels) by $N_\text {peaks}= 2^{N_\text {traps}}$. A more important factor to higher $\epsilon (\Delta _\text {RTS})$ is the influence of background noise size denoted as $Q_\text {wn}$, which is explicitly correlated to the spread of the peak distributions clearly seen in the Fig. 1b and the TLPs (Fig. 1c–g). In contrast to the examples of $Q_\text {wn}= 20\,\%$ in the leftmost column, the background noise completely hides individual peaks in the examples of $Q_\text {wn}= 100\,\%$ in the right column, producing a rather broad distribution in the TLPs that lowers the peak resolving accuracy. Surprisingly, despite the overwhelmingly strong background noise, $\epsilon (\Delta _\text {RTS})$ remains below ${2}{\%}$ for all three traps for $Q_\text {wn}\le {40}{\%}$. It is impressive that even for the worst class of $N_\text {traps}= 3$ and $Q_\text {wn}= {100}{\%}$, the third quartile sits below 10% error. This manifests that our KDE and GMM steps can acquire accurate estimates of $N_\text {traps}$ and $\Delta _\text {RTS}$ with confidence, which play a crucial role in the subsequent step where these values are used to generate tailored training data for our DNN model.

The third step aims to digitize RTSs accompanying background noise and to recover the pure RTS patterns. This process consists of several progressive tasks: differentiating RTS patterns from background noise, recording well-defined discrete states belonging to RTSs, setting decision criteria to assign each data point to one of the discrete states, and executing all time-series data in sequential or parallel manners. In validation, we quantify the digitization error $\epsilon (\eta )$ of the digitized RTSs at the third step with respect to the ground truth time series. Figure 3b–d plot $\epsilon (\eta )$ for all nRTSs as a function of $Q_\text {wn}$ with the trained Bi-GRU, Bi-LSTM, and WaveNet DNN models, respectively. Several common trends are captured in the $\epsilon (\eta )$ versus $Q_\text {wn}$ boxplots of all three DNN models. First of all, $\epsilon (\eta )$ is much higher than $\epsilon (\Delta _\text {RTS})$ in all examples, reflecting the difficulties in digitization. The behavior of $\epsilon (\eta )$ versus $N_\text {traps}$ and $Q_\text {wn}$ is expected: higher $\epsilon (\eta )$ for increased $N_\text {traps}$ and $Q_\text {wn}$ is similar to the $\epsilon (\Delta _\text {RTS})$ trend. However, the degradation in $\epsilon (\eta )$ is much more dramatic.

**Figure 4:**
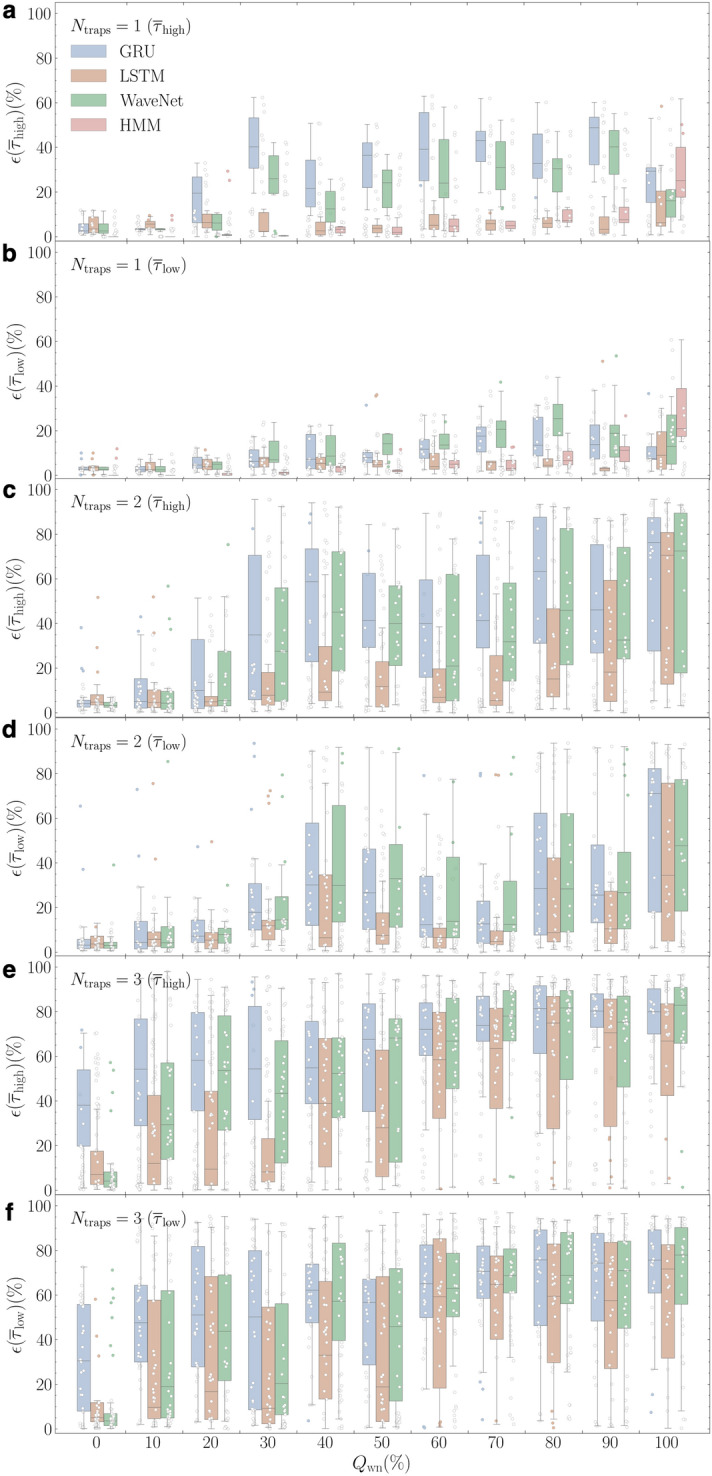
Comparison of two time-constant average errors from deep neural network models. $\epsilon (\bar{\tau }_\text {high})$ (**a**) and $\epsilon (\bar{\tau }_\text {low})$ (**b**) as a function of $Q_\text {wn}$ for all 110 examples from our nRTS white noise dataset where $N_\text {traps}$ = 1 for models Bi-GRU (blue), Bi-LSTM (orange), WaveNet (green), as well as HMM (red). $\epsilon (\bar{\tau }_\text {high})$ and $\epsilon (\bar{\tau }_\text {low})$ of $N_\text {traps}$ = 2 (**c**, **d**) and 3 (**e**, **f**) for three DNN models with the same color scheme.

From each digitized RTS time trace as a result of Step 3, we collect the histogram statistics of $\tau _{\text {high}}$ and $\tau _{\text {low}}$ from the fitting of Poisson function $f(t) = {\bar{\tau }}^{-1} \exp (-t/{\bar{\tau }})$ where a fitting parameter ${\bar{\tau }}$ yields $\bar{\tau }_\text {high}$ and $\bar{\tau }_\text {low}$. The ${\bar{\tau }}$ error ($\epsilon ({\bar{\tau }})$) is determined with the fitting values of ${\bar{\tau }}$ with synthetic RTS histograms. One thing we notice in the histograms of $\tau _{\text {high}}$ and $\tau _{\text {low}}$ with digitized RTSs is an abnormally high-intensity peak at the first bin far from the rest bins. We attribute it to false short-timed signals due to background noise components. Thus, since this peak yields shorter $\epsilon ({\bar{\tau }})$) not to represent true $\tau$ statistics, we exclude this distinct peak at the first bin for the Poisson function fit. In order to compare the results of three DNN models directly, we plot their $\epsilon (\bar{\tau }_\text {high})$ and $\epsilon (\bar{\tau }_\text {low})$ side by side given $N_\text {traps}$ in Fig. 4. For $N_\text {traps}$ = 1, we also include the HMM result after correcting the aforementioned flip error as a reference. In most $Q_\text {wn}$, HMM produces the smallest values of $\epsilon (\bar{\tau }_\text {high})$ and $\epsilon (\bar{\tau }_\text {low})$ against three DNN models; however, all DNN models outperform HMM, exhibiting lower error than HMM in the hardest case of $Q_\text {wn}= 100$%. Among the three DNN models, Bi-LSTM performance is noticeably better than the other counterparts for all $Q_\text {wn}$ except $Q_\text {wn}= 100$%. As the complexity of signals goes up $N_\text {traps}$ = 2 and 3 with bigger $Q_\text {wn}$, $\epsilon (\bar{\tau }_\text {high})$ and $\epsilon (\bar{\tau }_\text {low})$ become larger. Figure 4c and d plots the $\tau$-errors of $N_\text {traps}$ = 2, where the Bi-LSTM wins over the other two DNN models upto $Q_\text {wn}< 70$%. In the case of $N_\text {traps}$ = 2 with $Q_\text {wn}> 80$% and all $N_\text {traps}$ = 3 cases, the statistics of $\epsilon (\bar{\tau }_\text {high})$ and $\epsilon (\bar{\tau }_\text {low})$ look reasonably compatible.

### Validation on synthetic normal random telegraph signals with pink noise

In addition to white noise, we examine the effect of another common background fluctuation, pink noise, as a form of low-frequency noise. Its power spectral density (PSD) follows a trend of $1/f^\alpha$, where *f* is the frequency and $\alpha$ is the power-law exponent. We replace the previous Gaussian white noise with pink noise whose PSD follows the curve $1/f^{1}$. The pink noise magnitude in relation to the RTS magnitude is denoted $Q_\text {pn}$, which has a similar definition to $Q_\text {wn}$. For $Q_\text {pn}$ below 40 %, the influence of white noise and pink noise look alike, for example, in the cases of $Q_\text {wn}=$ 20 % in Fig. 1c and $Q_\text {pn}=$ 20 % in the left panel of Fig. 1f. However, higher $Q_\text {pn}$ RTSs (Fig. 1f, middle and right) display not only rapid wiggles but also slow-varying, incidental background trends that bury the RTSs immensely in the time domain. For the 330 RTS examples with pink noise, the same algorithm struggles both to accurately extract $N_\text {traps}$ and $\Delta _\text {RTS}$ in the KDE and GMM steps and to recover the pure RTS patterns in the digitization step.

**Figure 5:**
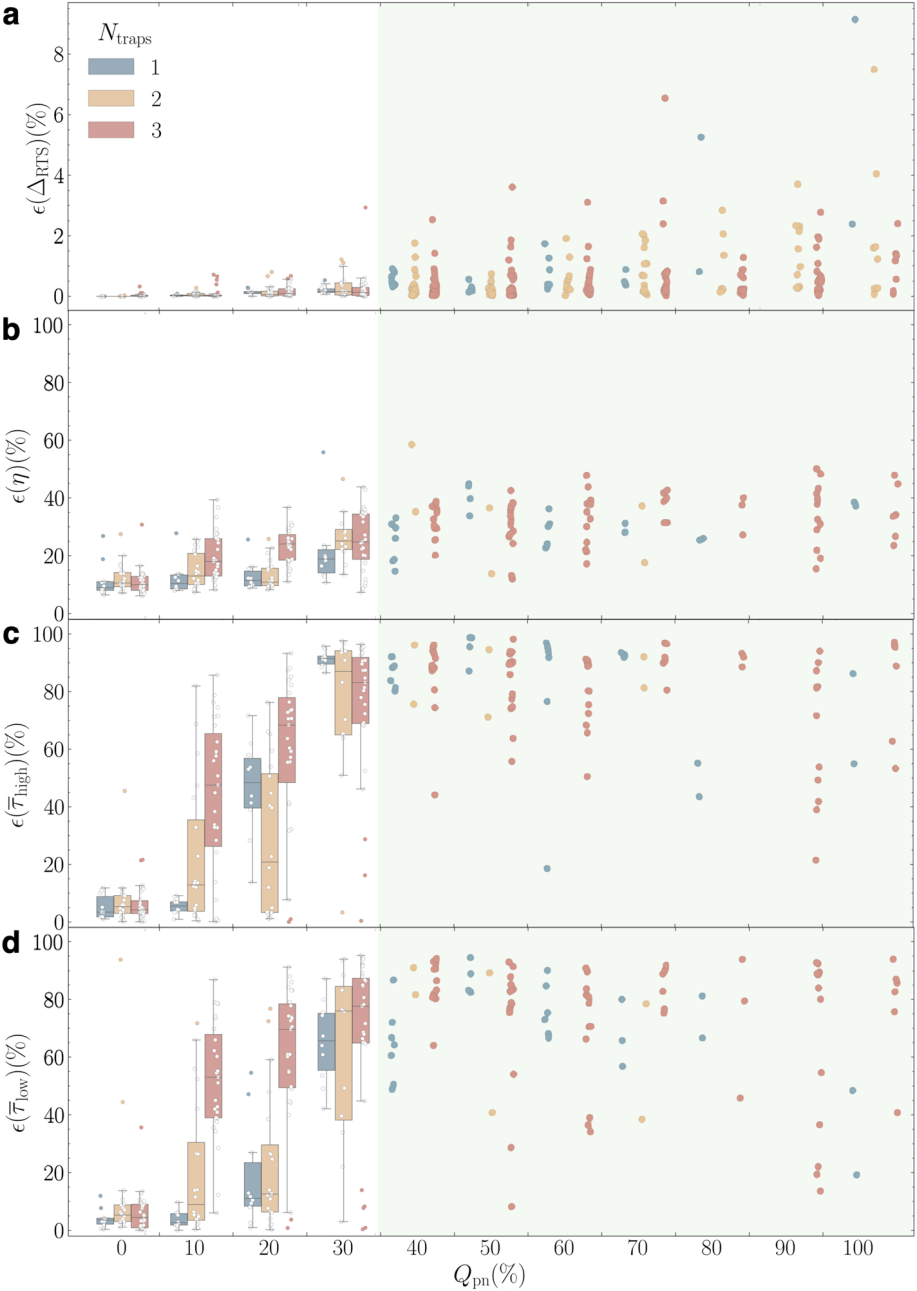
Validation results of the Bi-GRU model on normal RTS with pink noise. The errors in predicting $\Delta _\text {RTS}$ (**a**), the digitized signal (**b**), $\bar{\tau }_\text {high}$ (**c**), and $\bar{\tau }_\text {low}$ (**d**) against $Q_\text {pn}$ for $N_\text {traps}= 1$ (blue), 2 (yellow), and 3 (red) over our normal RTS dataset with pink noise. For $Q_\text {pn}> 30$%, the scattered plots are used since not all RTSs are identified successfully.

To analyze the pink noise effect on all 330 nRTS examples, we focus on the Bi-GRU RNN architecture which is fully connected and a newer RNN than the Bi-LSTM. When $Q_\text {pn}$ is less than 30 %, the correct number of traps is detected for all examples and $\epsilon (\Delta _\text {RTS})$ is in the same order as that of the white noise examples in the previous section. This means that our algorithm can recognize multilevel RTSs well for up to $Q_\text {wn}$ or $Q_\text {pn}$ = 30 % of both white and pink noise with 100 %, 95 %, and 92.5 % success probability of $N_\text {traps}$ = 1, 2, and 3, respectively in $Q_\text {pn}$ below 30%. On the other hand, if $Q_\text {pn} \ge 40\,\%$, we fail to accurately resolve all traps and their RTSs success probability is less than 60%, thus we use individual circles to represent data points instead of boxplots in Fig. 5. As long as the KDE and GMM steps find $N_\text {traps}$ and $\Delta _\text {RTS}$, the accuracies of both amplitude and digitization are as good as those of the white noise case, which is one important lesson. However, the mean of $\epsilon (\eta )$ is always higher than that of the white noise case, and the values of $\epsilon ({\bar{\tau }})$ in Figs. 5c and d soar up even with $Q_\text {pn}$ = 10%. We presume that if appropriate filtering to remove slowly-varying low-frequency behavior may reduce the pink noise effect, it may help to extract $\Delta _\text {RTS}$, $\bar{\tau }_\text {high}$ and $\bar{\tau }_\text {low}$ with better accuracy. We think this requires thorough studies with careful attention and filtering design since it is possible to filter short time periods accidentally to worsen the error by modifying statistics of time constants artificially^42^.

### Validation on synthetic anomalous random telegraph signals

**Figure 6:**
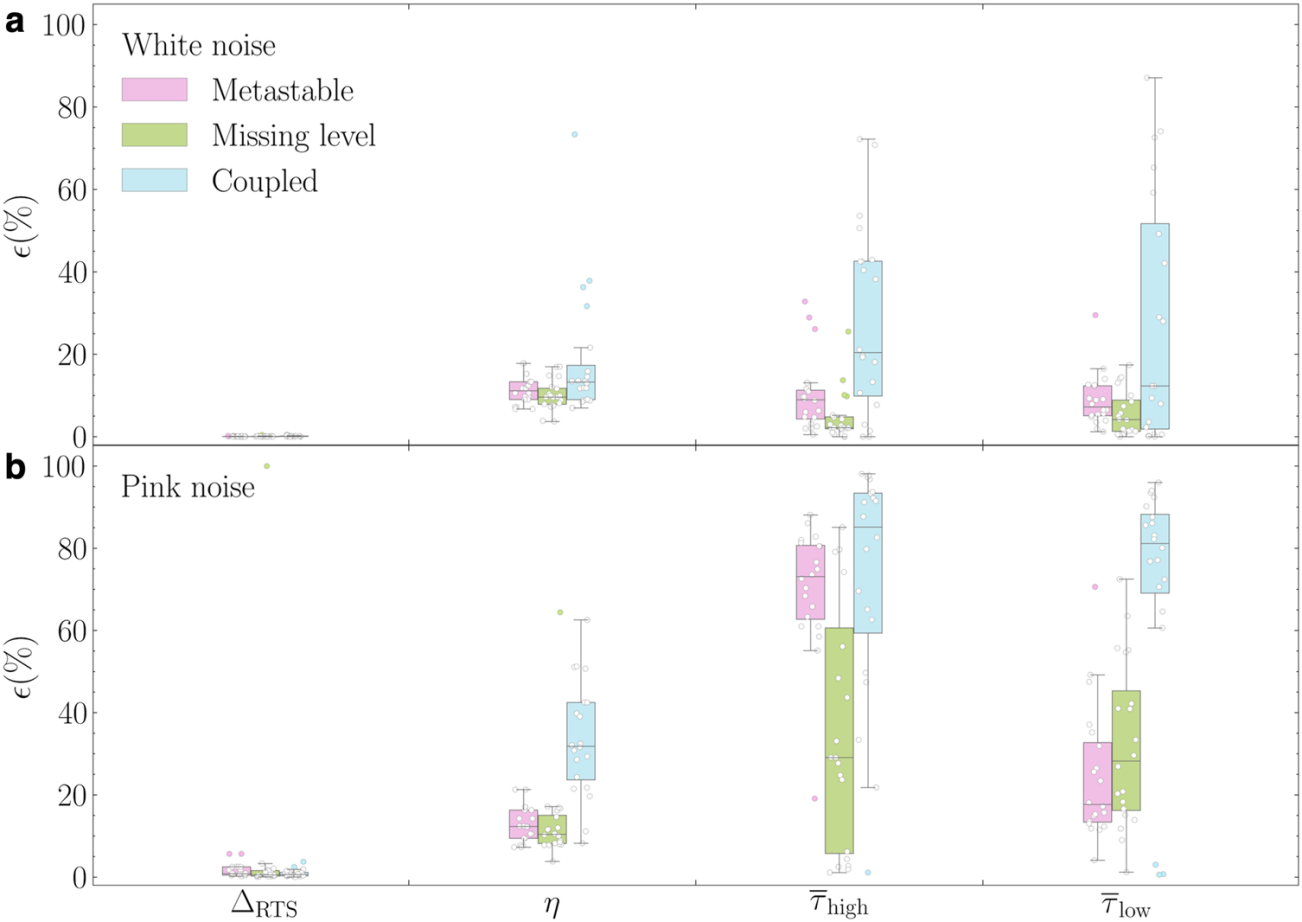
Validation results of the Bi-GRU model on anomalous RTS. (**a**) Errors of $\Delta _\text {RTS}$, $\eta$, $\bar{\tau }_\text {high}$ and $\bar{\tau }_\text {low}$ from three aRTS types degraded by white noise with $Q_\text {wn}$ = 20 %. (**b**) $\epsilon (\Delta _\text {RTS})$, $\epsilon (\eta )$, $\epsilon (\eta )$, and $\epsilon (\bar{\tau }_\text {low})$, where white noise is replaced by pink noise with $Q_\text {pn}= 20\,\%$.

We expand the validation of our algorithm further by testing it on aRTSs and present the results in Fig. 6. These signals in Fig. 1f may look somewhat similar to nRTSs, but there are some noticeable characteristics in time-series signals, often making aRTSs harder to address. Varieties of these aRTSs are grouped into three categories based on observable features^43,44^. The first group is labelled as “metastable” aRTS, where a reversal two-level RTS for $N_\text {traps}=1$ forms two contrasting zones A and B in the left of Fig. 1f. The origin is associated with a metastable state, which stays in the low state dominantly ($\bar{\tau }_\text {low}> \bar{\tau }_\text {high}$) during zone A, while the high state has longer dwell time ($\bar{\tau }_\text {high}> \bar{\tau }_\text {low}$) in zone B given the same trap. The other two categories are affiliated with multi-trap aRTSs, and we take $N_\text {traps}=2$ for demonstration. When two traps are strongly coupled, the switching of one trap depends exclusively on the state of the other. For example, one trap is only active when the other is also turned on. In this case, there are only three discrete levels instead of four, which is an example of “missing-level” aRTS (Fig. 1f, middle). The third type shows full four-level RTSs, but the switching amplitude $\Delta _\text {RTS}$ of each trap depends on the state of the other (Fig. 1f, right), whose category is named “coupled” aRTS.

For simplicity, we analyze 10 simulated signals of each aRTS type with fixed $Q_\text {wn}=~{{20}{\%}}$ as well as the same aRTS with $Q_\text {pn}={{20}{\%}}$ pink noise with the Bi-GRU. Figure 6a displays the results of this aRTS white noise study, where the mean amplitude extraction error is only 0.1%, which is comparable with the result from the nRTSs and $Q_\text {wn} = 20\,\%$. The digitization error is about 10-18 % on average compared to those of the nRTS counterpart, and the mean values of $\epsilon ({{\bar{\tau }}})$ are 7-10 % for the missing level and the metastable categories; however, the coupled case has the wide distribution of $\epsilon ({{\bar{\tau }}})$ and higher mean error $\sim 20$ %. The pink noise makes the aRTS problems harder as well with the higher error of $\Delta _\text {RTS}$, digitization, and two time-constants presented in Fig. 6b. Given this result from a limited dataset, we aspire to investigate aRTS thoroughly and to introduce a designated step for each aRTS type that can facilitate to establish an optimized algorithm in future work.

### Random telegraph signals of carbon nanotube films and semiconductor transistors

**Figure 7:**
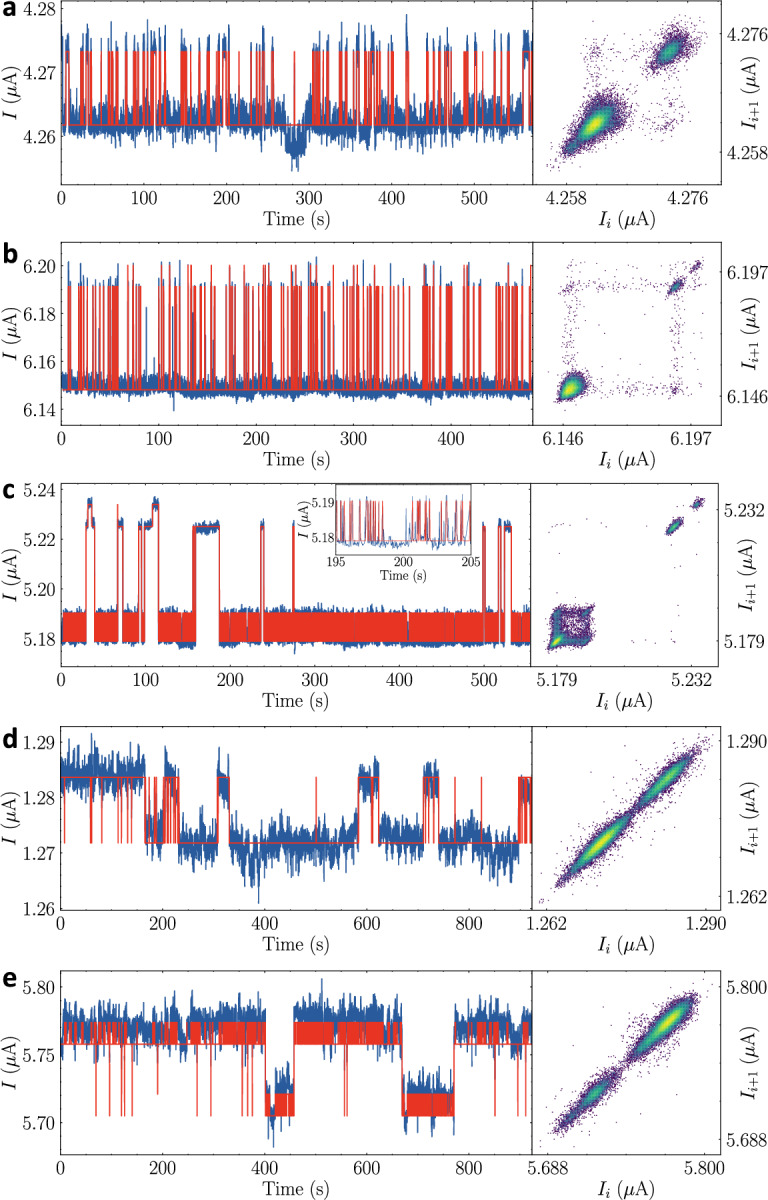
Analysis results of the Bi-GRU on real RTS data. In all five panels, the digitization results (red) are overlaid onto raw measurement data (blue) with a carbon nanotube film (**a**–**c**) and a short-channel 65 nm NMOS transistor (**d**, **e**) in the left panel and time-lag plots of the raw signal are given in the right panel. (**a**) We assign this signal as a two-level nRTS with low-frequency background noise. (**b**) An example of a missing level aRTSs is given. (**c**) Three-trap RTSs with different dwell time scales are seen as complicated aRTSs. The inset shows a zoomed-in view of the fast trap. (**d**) A two-level nRTS with pink noise is predicted and reconstructed. (**e**) Two-trap nRTS signals under pink noise background may be coupled in this trace.

The extensive validation tests on numerous synthetic RTSs grant confidence in our algorithm. However, this study would not be complete without examining real experimental data. A CNT device under tests has two terminals for voltage bias and a 65nm thick, 500nm wide multi-walled CNT film acts as a channel. The current through this channel is measured at 9K. In Fig. 7, three representative raw signals with increasing complexity are selected in blue, and overlaid in red are the digitized signals successfully obtained from our algorithm. Figure 7a and b are a simple two-level nRTS example and two mutually exclusive traps as a missing-level aRTS category, respectively. More complicated data are examined in Fig. 7c where aRTS-like features are observed. One small amplitude trap is only active when the large amplitude trap is inactive while the other small amplitude trap is only active when the large-amplitude trap is also active. Our analysis teaches that at least three traps are involved and some interactions among them exist. When we treat the data in Fig. 7c in the nRTS category, the resulting digitization was poor. Instead, we find that a missing-level aRTS analysis provides an extremely well-fitting digitization result. The other device is a NMOS, whose channel length is 65nm. The three-terminal device is placed at room temperature. Figure 7d shows a current sampling trace by applying a source-drain voltage at 10 mV and a gate voltage at 0.4 V. Our analysis algorithm predicts a reasonable fluctuating signal in an one-trap nRTS type with pink background noise. On the other hand, at another source-drain bias of 80 mV, the temporal current trace would resemble two-trap coupled aRTN type observed in Fig. 7e. The most dominant switching events occur in the higher two levels, and the low two levels do not show high frequencies. The overall shape of the signal looks similar to a 2-trap nRTS, but it may be predicted to be a coupled aRTS case depending on a context. Unlike the synthetic RTSs, the accuracy concept is not valid in real data because the ground truth values are not known; instead, our algorithm provides quantitative information on RTS parameters as a prerequisite for researchers to draw appropriate interpretations about their own systems.

## Discussion

The quantitative analysis of complex RTSs is essential to understand devices, systems, or processes in many scientific and engineering fields because they can reveal principal mechanisms and limit sensitivity. HMMs are extremely efficient to digitize two-level RTSs regardless of strong background noise, but their big issue is a binary flip error, namely, the labels of discrete two states are completely swapped, technically, 100 % error for around half of examples. While this flip error can be easily corrected in the simple case, it seems not to be straightforward for complex RTSs among many levels and level-couplings. In addition, the extension of HMMs or fHMMs into multi-level RTSs is not clear due to intrinsic uncertainty in sophisticated fHMM, which becomes a big hurdle to assess complex multi-level RTSs widely.

Our analysis protocol is structured in three steps and one of its key contributions is the progressive knowledge transfer approach, leveraging the knowledge discovered in early steps to generate tailored training data in the final step. A major advantage of our method over HMMs is that we predict the switching events of each trap independently, the superposition of which being the digitized multi-level RTS, whereas the latter predicts a single multi-level RTS. In the KDE and GMM steps, primary error sources are undesirable noise on top of RTSs. Therefore, $\epsilon (\Delta _\text {RTS})$ grows against $Q_\text {wn}$ and $Q_\text {pn}$. Strangely, we notice the non-negligible $\epsilon (\Delta _\text {RTS})$ in nRTS even for $Q_\text {wn}=0$ % and $Q_\text {pn}=0$ % which seem counter-intuitive. We attribute the $\Delta _\text {RTS}$ estimation error to the resolution of the KDE. Indeed, while the KDE is theoretically continuous, the implementation used operates finite input and output spaces, as is often the case for the computer implementations of mathematical concepts. The length and range of this space determine the finite resolution of the KDE in practice.

Numerous sources of error worsen digitization accuracy, consequently time-constant extraction accuracy, such as incorrectly assigning the RTS level between two close values, missing switching events, or adding false switching events. From time-bin histograms, as $Q_\text {wn}$ and $Q_\text {pn}$ go higher, very short false RTSs are more recurrent. The occurrence of these faults becomes more frequent in intricate, long-sequence RTSs with high $N_\text {traps}$ and background noise. Moreover, it is found that the information of neighboring data at prior and subsequent time steps can facilitate the decision of the state assignment. Recognizing RTSs as a sequence labeling task with the same input and output length, we approach ubiquitous RTS problems by resorting to powerful DNN architectures specifically designed for time-sequential data. Bi-GRU and Bi-LSTM RNNs are built and trained, where forward and backward propagation can include correlations of nearby data points to improve the analysis. We also examine another powerful network architecture known as Wavenet, a form of CNN^41^.

We summarize the training complexity of three DNN models in Table 1 (see “Methods”) and compare the training times of the three DNN models with a Titan Xp graphics card, 504 GB of memory, and 28 CPU cores. For $N_\text {traps}= 1$, we find that the simple HMM can solve each example within a duration of on average 1.8 s for $Q_\text {wn}$ = 0%, but around 20 s for $Q_\text {wn}$ = 100%, linearly increasing against $Q_\text {wn}$ values. Meanwhile, all DNN models take much longer than the HMM computation duration for $N_\text {traps}$ = 1. Thus, the HMM is a clear choice to examine two-level RTSs with the condition of the flip-error correction. However, we get an important observation that all three DNN models have reasonably constant training time regardless of $N_\text {traps}$, $Q_\text {wn}$, $Q_\text {pn}$, or the normal vs. anomalous cases. In terms of both the trainable parameters and training time, the CNN WaveNet model would be simpler and faster in the RTS analysis given our synthetic data. This should be further investigated whether this comes from the architecture difference between convolution layers and recurrent schemes. The insensitivity of the training time to parameters supports that the DNN models possess potential advantages and the capacity to handle much more complicated RTSs beyond our synthetic data.

In conclusion, we establish a structured methodology to analyze complex RTSs step by step with three DNN architectures, whose performance is directly validated with various types of RTSs and the effect of other background noise to collect the accuracy statistics of RTS parameters for the first time to our knowledge. We assert that our DNN-based algorithm is well-suited to characterize multi-level RTS in diverse fields, such as the study of nano- or micro-scale solid-state devices, quantum devices at cryogenic temperatures, chemical processes, and biological systems.

## Methods

### Systematic three-step algorithm

The algorithm is comprised of three major steps: first, the probability density of the raw signal is calculated and $N_\text {traps}$ is estimated; second, a GMM is fitted to the previous probability density to extract $\Delta _\text {RTS}$ of each trap; and third, these values of $N_\text {traps}$ and $\Delta _\text {RTS}$ are used to generated tailored training data for the DNN model to reconstruct the pure RTS.

*Step 1* Peak Estimation. The goal of this step is to recognize $N_\text {traps}$ present in the raw, noisy signal and to estimate the location of each peak in the probability density, which is a seed value for the GMM. To uncover peaks masked by white noise^45^, we first apply a moving average. Although the moving window width *M* is a tuning parameter, we successfully scale *M* by an estimate of the noise in the signal with the expression $M=\text {floor}[60 \cdot \tan ^{-1}(\sigma _{\text {wn}_\text {est.}} / 4) / \pi ]$, which gives excellent results over our dataset.

Then, we create a density distribution of the filtered data using KDE with a Gaussian kernel. Contrary to a histogram, the KDE is a continuously differentiable kernel function that leads to a smoother density distribution. Our Gaussian KDE reduces the number of false peaks caused by randomly fluctuating white noise, but maintains the ability to resolve close peaks.

*Step 2* Amplitude Extraction. In the presence of Gaussian noise, we can represent the probability density function (PDF) of an isolated RTS component by the mixture model of two Gaussian functions: $G_\text {high} = q \cdot G(\mu =I_0 + \Delta _\text {RTS}, \sigma )$ and $G_\text {low} = p \cdot G(\mu =I_0,\sigma )$ for high and low levels of the RTS, where $p = \frac{\bar{\tau }_\text {low}}{\bar{\tau }_\text {high}+ \bar{\tau }_\text {low}}$, $q = (1-p)$, and *G* is a standard Gaussian function with parameters mean ($\mu$) and standard deviation ($\sigma$). The total probability distribution $G_T$ for multi-trap RTS is constructed via the convolution of individual $G_\text {high}$ and $G_\text {low}$ pairs^46^. Finally, non-linear least-squares curve-fitting is applied to fit this model to the PDF obtained from the KDE. The fitted parameters of $G_T$ return the $\Delta _\text {RTS}$ of each trap.

*Step 3* Pure RTS Reconstruction. In this final step, the problem of reconstructing the masked RTSs is translated to a multi-output classification problem, which we solve with RNNs since these are well-suited for time-sequence data^47^. The input to the model is the time-sequence of a noisy RTS normalized between 0 and 1 and the outputs are time-series predictions for the state of each trap from the set ${\mathbb {S}} = \{\text {high},\text {low}\}$.

The RNN model is based on a 3-layer bidirectional gated recurrent unit (Bi-GRU) network^48,49^ with 256 neurons per layer (Fig. 1b). The input Bi-GRU layer takes historical information allowing a comprehensive analysis. With a window size of $W=64$, time steps 0–63 predict the RTS level at step 63, then the window is shifted to predict step 64 using steps 1–64, and so on. The last Bi-GRU layer yields a high-dimensional tensor including historical data. To obtain a single predicted RTS level per time step, this high-dimensional tensor is fed through a dense layer with softmax activation, whose output is reshaped to 3 dimensions sized $(W, N_\text {traps}, |{\mathbb {S}}|=2)$. Finally, the argmax function selects the most confidently predicted state $\in {\mathbb {S}}$ at each time step, and the desired time-series signal for each trap is acquired^50^.

RTS analysis is difficult to solve with supervised machine learning as there are no perfectly labelled measurement data with which to train. We solve this by training an RNN model for each example under test on twenty 50000-point synthetic RTS signals tailored for the specific $N_\text {traps}$, $\Delta _\text {RTS}$, and $\sigma _{\text {wn}_\text {est.}}$ which are quantified in previous steps. These data are shuffled and divided using a ${80}{\%}/{20}{\%}$ train/validation split.

In addition to GRU-based RNNs, we also test LSTM and WaveNet. In the case of LSTM, only the recurrent layers changed and the network architecture is otherwise identical. The WaveNet structure is created according to^41^. The number of trainable parameters and training time for each of these is given in Table 1 as an indicator for the architecture complexity.

**Table 1:** Training complexity of GRU, LSTM, and WaveNet architectures.

Architecture	#Trainable parameters	Training time
Bi-GRU	${695018} + N_\text {traps}\cdot {65792}$	${1350} {\textrm{s}} \pm {1} {\textrm{s}}$
Bi-LSTM	${922114} + N_\text {traps}\cdot {65792}$	${1570} {\textrm{s}} \pm {1} {\textrm{s}}$
WaveNet	${40130} + N_\text {traps}\cdot {8256}$	${897} {\textrm{s}} \pm {1} {\textrm{s}}$

The servers used for training the machine learning models are equipped with Titan Xp or Titan V graphics cards, 128-504  GB of memory, and 24-28 CPU cores.

### Generation of synthetic data

We synthesize 720 RTS examples in total and each example has a signal length of 1,000,000. Each RTS example has two components of (1) a digitized sequence between 0 and 1, and (2) background noise. First, a digitized sequence is produced by a Poisson distribution of a probability to dwell in the high and low states associated with random values of $\bar{\tau }_\text {high}$, and $\bar{\tau }_\text {low}$. This 0-1 digitized signal is multiplied by $\Delta _\text {RTS}$. Then, we generate background noise that follows Gaussian distribution. To make the synthetic data reflecting real situation, we consider both white noise and pink noise. The final synthetic data is formed by adding background noise to the RTS component.

### Deep neural network models

All of our codes are programmed in python platform, where we incorporate Tensorflow tools to build our DNN models using standard features of Keras layers including LSTM and GRU commands. The training epoch of our DNN model is 20, and we adopt the Adam as our gradient-based optimizer of stochastic functions^51^ and the learning rate of the optimizer is 0.0001. The loss function used for digitization is the cross-entropy function, which is calculated by,

$$\begin{aligned} L_{ce} = - \sum ^N_{n=1} t_n \log p_n, \end{aligned}$$

where *N* is the number of classes, $t_n$ is the true label of *n*-class and $p_n$ is a softmax probability of the class *n*.

## Data Availability

The datasets used and/or analysed during the current study are available from the corresponding author upon reasonable request.
